# Genome sequence of a monoterpene metabolizing strain *Sphingobium yanoikuyae* A-TP

**DOI:** 10.1128/mra.00233-26

**Published:** 2026-06-04

**Authors:** Nemanja Jankovic, Giovanni Marques de Castro, André Luiz Quintanilha Torres, Rafael Chelala Moreira, Marcela Uliano-Silva, Cinara Souza da Conceição, Vitor Lima Coelho, Juliana Alves Americo, Juliano Lemos Bicas, Mauro de Freitas Rebelo

**Affiliations:** 1 Carlos Chagas Filho Institute for Biophysics, Federal University of Rio de Janeiro 28125, Rio de Janeiro, Brazil; 2 Department of Innate Immunity, UMass Chan Medical School 12262, Worcester, Massachusetts, USA; 3 Department of Entomology, University California 385623 , Riverside, California, USA; 4 Department of Food Science and Nutrition, School of Food Engineering, State University of Campinas 534751, Campinas, Brazil; 5 Tree of Life, Wellcome Sanger Institute, Cambridge, United Kingdom; 6 Faculty of Biosciences and Aquaculture, Nord University 168026, Bodø, Norway; 7 Firjan SENAI, Rio de Janeiro, Brazil; 8 Laboratory of Stem Cells and Bone Regeneration, Federal University of Rio de Janeiro 28125, Rio de Janeiro, Brazil; 9 Bio Bureau Biotechnology, Rio de Janeiro, Brazil; Rochester Institute of Technology, Rochester, New York, USA

**Keywords:** biotechnology, monoterpenes, *Sphingobium*, gene discovery

## Abstract

Here, we report the draft genome sequence of the *Sphingobium yanoikuyae* A-TP (formerly *Pseudomonas fluorescens* NCIMB 11671) strain that can grow on ɑ-pinene and limonene. The draft genome of this strain is 5.6 Mb and revealed two ~30-kb gene clusters putatively involved in the degradation of ɑ-pinene, limonene, and citronellol.

## ANNOUNCEMENT

Monoterpenes are key fragrance precursors and ingredients (1). Deciphering the degradation pathways of monoterpenes by microorganisms is important because it can lead to more efficient production of these compounds and their derivatives.

*Sphingobium yanoikuyae* from this study was isolated as *Pseudomonas fluorescens* NCIMB 11671 from activated sludge using ɑ-pinene as a sole carbon source (2). It was later reclassified as *S. yanoikuyae* (3) and is referred to as A-TP strain in this report. This strain was found to grow on monoterpenic substrates such as limonene, ɑ-pinene, and β-pinene (4), and it harbors an additional energy and co-factor independent pathway by which it converts limonene to ɑ-terpineol (5, 6). We sequenced the genome of A-TP strain to gain better understanding of the genetic determinants that underlie its ability to utilize these industrially important compounds.

The total DNA for genomic sequencing was prepared from 1 mL of fresh A-TP inoculum, grown on Tryptic soy broth overnight at 200 rpm at 30°C, isolated with the DNeasy Blood & Tissue DNA kit (QIAGEN) according to the supplier’s instructions and stored in TE buffer (10 mM Tris 1 mM EDTA, pH 8.0). The DNA was lyophilized and sent to GenOne: Soluções em Biotecnologia (Rio de Janeiro, RJ, Brazil) for sequencing of 2 Gb (Q30 84.1%) with 250-bp paired-end sequencing on Illumina HiSeq 2000 platform, with default parameters. About 1.5 μg of enzymatically digested DNA was used to prepare sequencing libraries whereby the fragments were end-repaired, A-tailed, and ligated using NEB Next Ultra DNA Library Prep Kit, according to the manufacturer’s recommendations, yielding 5,299,754 raw reads.

**Fig 1:**
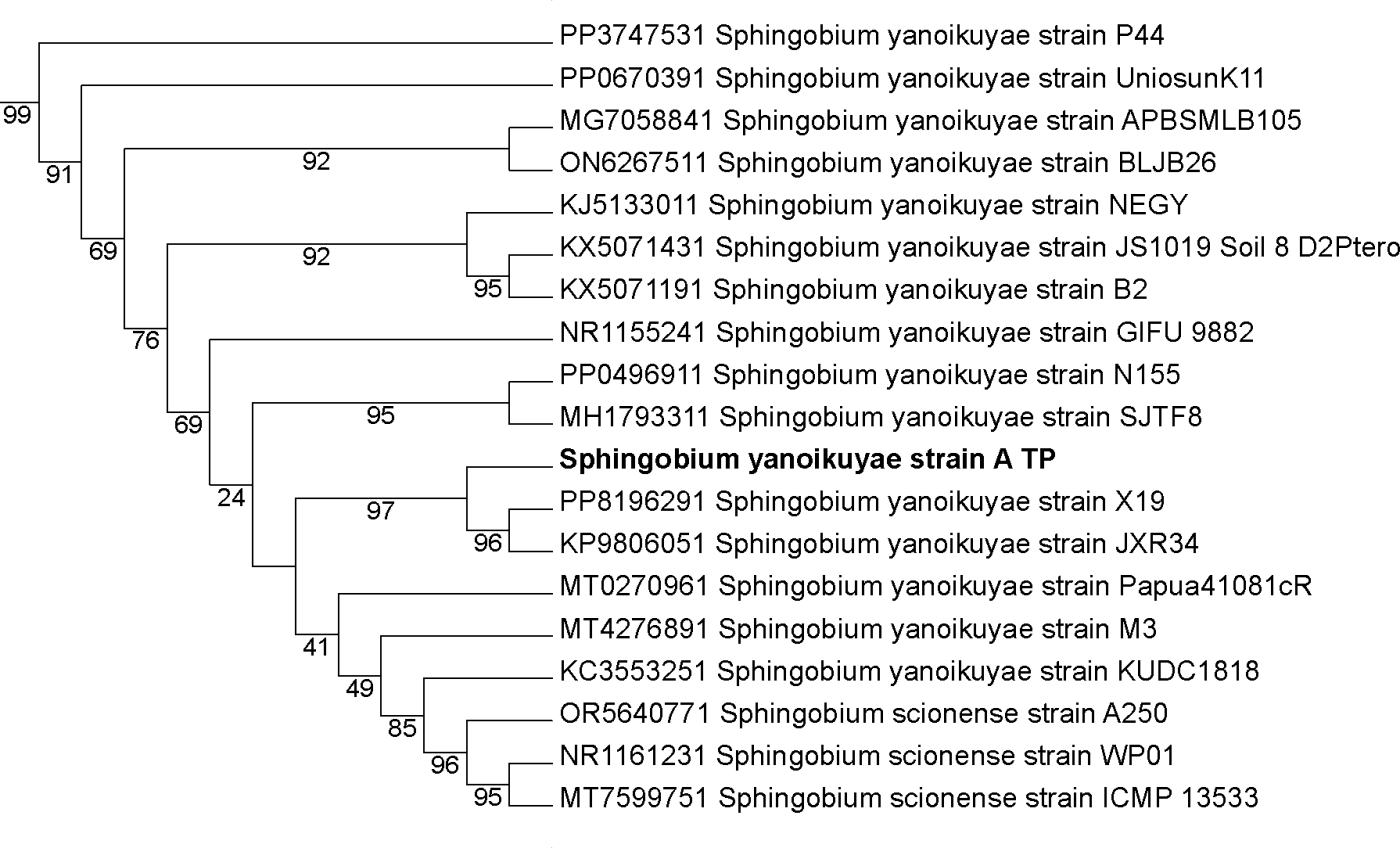
The maximum likelihood tree of 53 *Sphingobium* species and 15 *S. yanoikuyae* strains, with the A-TP strain in bold. The sequences were aligned with MAFFT (11) and manually curated for reliability. The tree was constructed using IQ-TREE2 (12), with the evolutionary model selected for optimal fit (K2P + I + G4), branch support assessed with 10,000 ultrafast bootstrap replicates, and visualized with the iTOL (13).

Read quality was analyzed with the Fastqc v.0.11.5 (7), adapters were removed via fastp v1.0.1 (https://doi.org/10.1002/imt2.107), and the genome was assembled with SPAdes v.3.15 (8) (parameters --careful --cov-cutoff auto). The corresponding genes and proteins were predicted and functionally annotated with RASTtk within the BV-BRC platform (v.3.55.17) (9). BUSCO (v.6.0.0) analysis revealed the presence of 100% of the orthologous genes conserved in bacteria, indicating a satisfactory genome assembly and protein prediction (10). The draft genome consists of 98 contigs, with an N_50_ of 199,910 bp and a total size of 5,571,158 bp (Table 1). Phylogenetic analysis of 16S rRNA gene showed that the A-TP formed a single clade with other *S. yanoikuyae* strains, of which X19 and JXR34 isolates (bootstrap support of 97) are indicated as the most recent common ancestors (Fig. 1).

**TABLE 1:** Genome features of *Sphingobium yanoikuyae* A-TP

Feature	Result
Genome length (bp)	5,571,158
Contigs	98
GC content (%)	64.0
Total genes	5,593
Protein-coding genes	5,526
rRNA genes	9
tRNA genes	58
Scaffold N50 (bp)	199,910
Average fold coverage (×)	444.1
NCBI WGS accession number	JBJYKA020000000
NCBI BioProject accession number	PRJNA1196986
NCBI BioSample number	SAMN45767236

Genome annotation revealed two ~ 30-kb clusters that contain putative genes for initial degradation of limonene, ɑ-pinene, and citronellol, among which are those that share between 22% and 68% similarity to those reported in other monoterpene-metabolizing microorganisms, such as *Rhodococcus erythropolis* DCL14 (AJ272366.1), *Pseudomonas rhodesiae* CIP 107491 (MF946559.1), *Pseudomonas aeruginosa* PAO1 (AE004091.2), and *Grosmannia clavigera* kw1407 (GL629729.1, NW_014040759.1). The findings provide a solid basis for experimental validation of the proposed gene functions regarding degradation of monoterpenes and their derivatives by this industrially important strain.

## Data Availability

This Whole Genome Shotgun project has been deposited in DDBJ/ENA/GenBank under accession number JBJYKA000000000. The version described in this paper is version JBJYKA020000000. The project data are available under BioProject accession number PRJNA1196986, Sequence Read Archive under accession number SRR31950839, and BioSample under accession number SAMN45767236.
